# A Professional Warning

**Published:** 1897-06

**Authors:** Mark G. M'Elhinney

**Affiliations:** Ottawa, Ont.


### Article IX.

### A PROFESSIONAL WARNING.

BY MARK G. M'ELHINNEY, D. D. S., L. D. S., OTTAWA, ONT.

Read before the Ottawa Dental Association, Jan. 12th, '97.

The professions of medicine and dentistry are divided into two great classes, legitimate practitioners and quacks. Each of these professions, as the result of long experience, has accumulated a set of rules, written and unwritten for the guidance of its members. These rules enjoin those lines of conduct that will conduce to the best interests of the practitioners, and ensure protection and the highest class of service to the public.

Those members of a profession who observe the the spirit of these rules are looked upon as legitimate practitioners. Those who disregard the rules are called quacks. Nearly all start out in the legitimate line, but bad fortune, inability to face the long and weary struggle of practice building, bad counsel or example, desire to grow rich rapidly without considering the means, and many other causes weed out a percentage and send them forth to prey upon the world as quacks. Very few quacks succeed in making a brilliant success, for it requires a superlatively clever man to fool the public for any great length of time—the remainder of these sink lower and lower until they become mere street-corner fakirs. The quack holds the same relation to the legitimate practitioner that the tinker does to the skilled engineer, the scab to the honest skilled laborer, and the tramp to the reputable citizen. The quack is a professional tramp. The quack may be, in fact frequently is, a skillful man; there have been Napoleonic fakirs in all lines of life, but his methods are not those best calculated to ensure the best welfare of his patients.

There are several reasons why itinerant practitioners, even if skilful, cannot give as good services as regular practitioners. The itinerant has no fixed place of business, no regularly equipped surgery and laboratory. He must put up with the various inconveniences and annoyances of temporary quarters, consequently his services must vary with his circumstances. The quack, in moving from place to place, trusts to his advertising for patronage and does not gain permanent patients, hence it matters little to him what may be the result of his work, for he has no reputation to preserve or character to protect. The quack often, nay, nearly always lays claim to skill far beyond the range of the regular dentist or physician. A moment's reflection will show that a person possessing such superior skill and powers could attain fame and fortune legitimately in any great city and must be a fool to turn peddlar and give his services at half price. The quack having obtained his dupes' money, wishes quickly to be rid of them, and does not care what future trouble may ensue, hence it is positively dangerous to engage the services of such totally irresponsible persons. A case in point—several years ago a dentist came to Ottawa equipped with a brass band, a variety troupe and electric light. He sold patent medicines and extracted teeth by the thousands. Between the distracting noise of the band and the effect of a powerful drug contained in a bulb on the handle of the forceps he actually extracted a large number of teeth in a painless manner. What was the result? A large proportion of the teeth were broken in the rapidity of the operation, the victim being hustled down too quickly to discover this or make protest. A large number of good teeth were sacrificed, the extraction of which was little short of criminal. The last and most serious result was the subsequent inflammation and sloughing caused by the drug or some of its constituents. There were many cases of serious inflammation and suppuration, and in at least one case the victim was in danger of losing the whole lower jaw through caries. Such are the unlovely results of such wholesale malpractice.

The claims of these men to superior skill and special knowledge are totally unfounded. Each and every dentist in Ontario must attend the Royal College of Dental Surgeons for the full course and must pass the prescribed examinations. The opportunities for instruction are equally open to every student. Every effort is made by the professors to obtain the very latest methods and most modern appliances, and no improvement can exist for any length of time without its advent becoming a matter of discussion in the dental journals, which are widely circulated and read by professors, students and practitioners. There are no trade secrets in dentistry. Individual variations in method are many, and of these any practitioner may choose that best fitted to his requirements, but the system of modern dentistry in all its intricacy is the common property of the dental profession. Our surgeries and laboratories are ever open to our brother practitioners, and at our meetings together we exchange and discuss our ideas and opinions, and above all, what characterizes most dentists as men of liberal and scientific spirit, is that any one who makes an improvement or discusses a new fact immediately calls his brethren together and gives them the benefit of his good fortune. Is it reasonable to believe that an itinerant tooth-puller could by any possibility possess knowledge that could remain hidden from a numerous and educated profession that singly and collectively is continually striving for higher and better attainments?

The claims of the quack are based as much upon his own ignorance as upon his knowledge of the gullibility of human nature. It was not long ago that a young man called at the writer's surgery and endeavored to sell a secret method in connection with crown and bridge work. He was surprised to find that the writer had been shown the same method by another older practitioner in this city, and that in all probability the method is in the possession of almost every member of the profession to-day. In addition, the method is one of minor importance, suited only to occasional cases and more a trick of manipulation than any real improvement in result.

The possession of the degree in dentistry is sufficient evidence of average ability, which is all that average recompense calls for. It may be held that the regular practitioner is afraid that he will lose money if the public patronize itinerant operators. There is also something in this, for the resident practitioner pays rent and taxes, and otherwise contributes to the general prosperity of the community, and the better the public supports him the better services he can render. Every professional man spends much time and money in adding to his knowledge regarding difficult points in practice, and frequently on an especially difficult case he may spend ten times as much as that case can repay. The writer is aware of more than one case where the practitioner, for the sake of the patient, for the sake of his own reputation, and for his own professional satisfaction, brought it to a successful termination at an expense not only far beyond anything received in fees, but owing to fewness of such cases beyond any possibility of future remuneration.

It has been charged that we labor only for the almighty dollar. Were it not for necessity it is probable that many of us would not be in the profession; but once in, it is a poor and mean individual who does not develop some interest in the welfare and advancement of his chosen calling. Cheapness is the great bait held out by quackery. Mean and ignorant persons imagine that they have done a smart thing in saving a few dollars in medical fees, even if they have, by resorting to quackery, lost the life of a wife or child. It is the same in dentistry. The same people will stop at half a dollar in the price of a filling, even when it may mean the loss of a tooth which is a vital necessity to health and comfort. Even if the quack rendered equally efficient services, which cannot for a moment be admitted, it is a manifest injustice to extend to him the patronage that rightly belongs to the resident practitioners, for to them all other members of the community are more or less indirectly indebted as members of the same social arrangement.

In some of the smaller towns and villages where the population of each is not sufficient to support a resident dentist, it is necessary for one to visit them at intervals, but this practitioner always has an established headquarters and does not come under the definition of a quack. Some of the most reliable men in the profession have practice of this description. They have a clientele in each place, and are as careful of their pofessional reputation as any of the resident dentists of a large city. The advent of a quack always results in the regular dentists being crowded by a lot of difficult and unsatisfactory cases for which equivalent remuneration is very difficult to obtain. The cases consist of misfitting artificial teeth, uncleanly crowns, roots left in during careless extraction, inflammation from use of powerful drugs, and sometimes even fracture of the jaw from brutal manipulation, and blood-poisoning from the use of uncleansed instruments. Imagine for a moment the extraction of hundreds of teeth in an evening without even wiping the forceps, as the writer has actually seen take place. What a splendid op portunity for the wholesale dissemination of syphilis and kindred diseases. This sort of thing is criminal, and should entitle the perpetrator to a term of imprisonment that would teach him not to indulge in malpractice. The serious results that may follow the use of unclean surgical instruments are not half appreciated by the laity. Ask any physician, and he will say that since the advent of antiseptic surgery the mortality from surgical operations has decreased to an astonishing degree.

It is a fact that the existence of syphilitic virus in the first of one hundred patients operated upon consecutively with unclean forceps could entail life-long misery upon the other ninety-nine, not to speak of transmitted disease through countless generations of their posterity. The dentist who for a greatly reduced fee is compelled to handle too many victims in a given time to make a living, has not time left to attend to such a small, though important, matter as the cleaning of instruments. He is forced to leave the debris, and perhaps deadly poison, of the last operation in the mouth of the patient next following.

There is no doubt that a restaurant keeper who took no time to wash dishes and table linen could provide meals for a few cents less, but how many customers would he get even in the slums ? Yet in a matter of a thousand times more importance some people, for that few cents, will run the risk of misery and even death itself.

A great many would-be wise persons refuse to be warned that the services of the cheap john are most expensive in the end, and like the people described in the legend of the Noachian deluge, keep r ght on to destruction. Be that as it may, the profession, in sounding many a note of warning, has the satisfaction of knowing that it has, as far as lay in its power, done its duty.—*Dominion Dental Journal.*

